# The agreement chart

**DOI:** 10.1186/1471-2288-13-97

**Published:** 2013-07-29

**Authors:** Shrikant I Bangdiwala, Viswanathan Shankar

**Affiliations:** 1 Department of Biostatistics, Gillings School of Global Public Health, University of North Carolina, Chapel Hill, NC 27599, USA; 2 Division of Biostatistics, Department of Epidemiology and Population Health, Albert Einstein College of Medicine, Bronx, NY, USA

**Keywords:** Intra- and inter-observer agreement, Concordance, Kappa statistic, B-statistic

## Abstract

**Background:**

When assessing the concordance between two methods of measurement of ordinal categorical data, summary measures such as Cohen’s (1960) *kappa* or Bangdiwala’s (1985) B-statistic are used. However, a picture conveys more information than a single summary measure.

**Methods:**

We describe how to construct and interpret Bangdiwala’s (1985) agreement chart and illustrate its use in visually assessing concordance in several example clinical applications.

**Results:**

The agreement charts provide a visual impression that no summary statistic can convey, and summary statistics reduce the information to a single characteristic of the data. However, the visual impression is personal and subjective, and not usually reproducible from one reader to another.

**Conclusions:**

The agreement chart should be used to complement the summary kappa or B-statistics, not to replace them. The graphs can be very helpful to researchers as an early step to understand relationships in their data when assessing concordance.

## Background

When two raters independently classify the same n items into the same k ordinal categories, one wishes to assess their concordance. Such situations are common in clinical practice; for example, when one wishes to compare two diagnostic or classification methods because one is more expensive or cumbersome than the other, or one wishes to assess how well two clinicians are in blindly classifying patients into disease likelihood categories.

### Example 1

In Landis & Koch [1], the authors review an earlier study of the diagnosis of multiple sclerosis by Westlund & Kurland [2], where investigators were interested in the possibility that the disease was distributed differently geographically. They studied a series of 149 patients from Winnipeg, Manitoba, Canada, and a series of 69 patients from New Orleans, Louisiana, USA. Both sets of patients were classified independently by both sets of neurologists, after they were requested to disregard their original diagnosis, into four diagnostic categories – certain, probable, possible and ‘doubtful-unlikely-definitely not’ multiple sclerosis. The resulting tabulations are in Table 1.

**Table 1:** **Cross tabulations of multiple sclerosis diagnosis by two independent neurologists, comparing concordance with different sets of patients - [Westlund** &**Kurland (1953)]**

		**(A) Winnipeg patients**					**(B) New Orleans patients**				
		**Winnipeg neurologist**					**Winnipeg neurologist**				
		**Certain**	**Probable**	**Possible**	**No**	**Total**	**Certain**	**Probable**	**Possible**	**No**	**Total**
New Orleans neurologist	Certain	38	5	0	1	44	5	3	0	0	8
	Probable	33	11	3	0	47	3	11	4	0	18
	Possible	10	14	5	6	35	2	13	3	4	22
	No	3	7	3	10	23	1	2	4	14	21
	Total	84	37	11	17	149	11	29	11	18	69

One can assess concordance between the neurologists naively by calculating the proportion of observations in the diagonal cells; but more commonly, one uses either Cohen’s [3] kappa statistic or Bangdiwala’s [4] B-statistic, both of which account for chance agreement. The choice between and interpretation of these two statistics was reviewed in Muñoz & Bangdiwala (1997) [5] and Shankar & Bangdiwala (2008) [6], which also discusses the methodology behind both statistics.

One can account for partial agreement by considering the weighted versions of these two statistics, which assign weights to off-diagonal cell frequencies in their calculations. We considered quadratic weights for calculating weighted statistics in this manuscript. For Table 1A, the Winnipeg patients, the statistics are kappa = 0.208 (weighted kappa = 0.525) and B = 0.272 (weighted B = 0.825), while for Table 1B, the New Orleans patients, the statistics are kappa = 0.297 (weighted kappa = 0.626), and B = 0.285 (weighted B = 0.872). These values would be considered as ‘fair’ to ‘moderate’ but they are not meaningfully different between Winnipeg and New Orleans patients.

### Example 2

In the Lipids Research Clinics Program Mortality Follow-Up Study (LRC-FUS), all deaths were classified by a trained nosologist, but all deaths suspected to be related to cardiovascular disease were also classified following a rigorous, lengthy, cumbersome and expensive review by an expert panel of cardiologists [7]. Of interest was to assess whether the more expensive process was necessary, by examining the concordance of both measurement methodologies, with special attention to deaths in elderly (≥65 years) versus non-elderly (<65 years) deaths, focusing on whether they were cardiovascular or non-cardiovascular. The resulting tabulations are in Table 2.

**Table 2:** Cross tabulations of cardiovascular disease cause of death by two independent classification methodologies in the lipids research clinics program mortality follow-Up study (LRC-FUS), comparing elderly (≥65 years) and non elderly deaths – [Bangdiwala et al. (1989)]

		**(A) Elderly**			**(B) Non-elderly**		
		**(≥65 years) deaths**			**(<65 years) deaths**		
		**Nosologist**			**Nosologist**		
		**CVD**	**Non-CVD**	**Total**	**CVD**	**Non-CVD**	**Total**
Expert panel	CVD	172	11	183	122	10	132
	Non-CVD	35	50	85	5	18	23
	Total	207	61	268	127	28	155

For Table 2A, the elderly deaths, the summary concordance measures are kappa = 0.57 and B = 0.74, while for Table 2B, the non-elderly deaths, these measures are kappa = 0.65 and B = 0.87. These can be interpreted according to Muñoz & Bangdiwala [5] as ‘substantial’ to ‘almost perfect’ agreement, but they are not meaningfully different between elderly and non-elderly deaths.

### Example 3

In a recent article in *BMC Cancer*, Garrido-Estepa et.al. [8] compared four classification scales of risk categorizations for breast cancer based on mammographic density patterns from women included in the DDM-Spain study. Each of 375 randomly selected mammograms was read twice by an expert radiologist and classified using the Wolfe, Tabár, BI-RADS and Boyd scales. The resulting cross-tabulations are in Table 3, along with a description of the various scales.

**Table 3:** Cross-tabulations of numbers of mammograms according to risk categories for breast cancer, comparing concordance among scales of mammographic density patterns - [Garrido-Estepa et al. 2010]

		**(A) Wolfe classification scale**						**(B) Tabár classification scale**						
		**Second measure**						**Second measure**						
		**N1**	**P1**	**P2**	**DY**	**Total**		**II**	**III**	**IV**	**V**	**Total**		
First measure	N1	12	9	0	0	21	II	12	9	0	0	21		
	P1	4	139	13	5	161	III	4	170	16	8	198		
	P2	0	7	101	14	122	IV	0	4	114	6	124		
	DY	0	2	13	56	71	V	0	8	9	15	32		
	Total	16	157	127	75	375	Total	16	191	139	29	375		
		**(C) BI-RADS classification scale**					**(D) Boyd classification scale**							
		**Second measure**						**Second measure**						
		**AEF**	**SFD**	**HD**	**ED**	**Total**		**A**	**B**	**C**	**D**	**E**	**F**	**Total**
First measure	AEF	147	13	0	0	160	A	6	4	0	0	0	0	10
	SFD	14	101	10	0	125	B	4	56	11	0	0	0	71
	HD	0	14	48	6	68	C	0	16	50	13	0	0	79
	ED	0	0	3	19	22	D	0	0	14	102	9	0	125
	Total	161	128	61	25	375	E	0	0	0	14	48	6	68
							F	0	0	0	0	3	19	22
							Total	10	76	75	129	60	25	375

(A) **Wolfe:**

Low-risk categories:

N1: Breast composed almost completely of fat, with perhaps just a few fibrous connective tissue strands.

P1: Breast composed mainly of fat, although up to a quarter of the sub-areolar area may show beaded or cord-like areas corresponding to prominent ducts.

High risk categories:

P2: More severe involvement of the breast, with a prominent duct pattern occupying more than one quarter of breast volume.

DY: Breast typically contains extensive regions of homogeneous mammographic densities, which appear as sheet-like regions. The proportion of density is greater than that of the fat.

(B) **Tabár:**

Low risk categories:

I: Mammogram composed of scalloped contours with some lucent areas of fatty replacement and 1 mm evenly distributed nodular densities.

II: Mammogram composed almost entirely of lucent areas of fatty replacement and 1-mm evenly distributed nodular densities.

III: Prominent ducts in the retroareolar area.

High risk categories:

IV: Extensive, nodular and linear densities with nodular size larger than normal lobules.

V: Homogeneous ground-glass-like appearance with no perceptible features.

(C) **BI-RADS:**

Low risk categories:

Almost entirely fat: 0-25%.

Scattered fibroglandular densities: > 25-50%.

High risk categories:

Heterogeneously dense: > 50-75%.

Extremely dense: > 75%.

(D) **Boyd:**

Low risk categories:

A: 0%.

B: > 0-10*%.

C: 10-25*%.

D: 25-50*%.

High risk categories:

E: 50-75*%.

F: ≥75%.

*Upper bound excluded.

The reported values of agreement for the various scales between the first and second readings were 0.73, 0.72, 0.76 and 0.68 for the kappa for the Wolfe, Tabár, BI-RADS and Boyd scales, respectively, and 0.71, 0.75, 0.74 and 0.58 for the B-Statistic for the same scales. The kappa statistics and B-statistics fall into Muñoz & Bangdiwala’s interpretations between ‘substantial’ to ‘almost perfect’, indicating great concordance, but with no meaningful differences among the 4 classification scales with respect to concordance between first and second measure. In Table 3 we note that discrepancies for the BI-RADS and the Boyd classifications are only for contiguous risk categories, while for Wolfe and Tabár they sometimes are two risk categories apart. The weighted versions of the statistics [kappas of 0.84, 0.71, 0.90 and 0.92 for the Wolfe, Tabár, BI-RADS and Boyd scales, respectively, and B-Statistics of 0.96, 0.95, 0.97 and 0.98 for the same scales] are thus much closer to unity than the un-weighted versions.

The above three examples illustrate the wide need for assessing agreement in the clinical field, and the utility of alternative summary statistics. While both statistics can summarize the agreement information numerically, however, a graph can ‘tell a story’. The agreement chart [4] is a two-dimensional graph for visually assessing the agreement between two observers rating the same n units into the same k discrete ordinal categories.

## Methods

### Constructing the agreement chart

The agreement chart is a visual representation of a k × k square contingency table. It is constructed with the following steps:

i Draw an n × n square.

ii Draw k rectangles of dimensions based on the row and column marginal totals, placed inside the n × n square, and positioned with the lower left vertex touching the upper right vertex of the previous rectangle, starting from the (0,0) position to the (n,n) point of the large square.

iii Draw k shaded squares of dimensions based on the diagonal cell frequencies, placed inside the corresponding rectangle, positioned based on the off-diagonal cell frequencies from the same row and column.

iv ‘Partial agreement’ areas can be similarly placed within the rectangles, with decreasing shading for cells further away from the diagonal cells.

The statistical software SAS version 9.3 has incorporated the agreement plot as a default chart (see PROC FREQ under AGREE and KAPPA syntax). The agreement chart is also implemented in the open-access R software under the vcd package [9]. The agreement chart provides a visual representation for comparing the concordance in paired categorical data. The visual image is affected if the order of the categories is permuted, and thus its use is recommended exclusively for ordinal level variables. In the case of perfect agreement, the k rectangles determined by the marginal totals are all perfect squares and the shaded squares determined by the diagonal cell entries are exactly equal to the rectangles, producing a B-statistic value of 1. Lesser agreement is visualized by comparing the area of the blackened squares to the area of the rectangles, while observer bias is visualized by examining the ‘path of rectangles’ and how it deviates from the 45° diagonal line within the larger n × n square.

## Results

### Examples with charts

#### Example 1 - Westlund & Kurland (1953) multiple sclerosis

**Figure 1:**
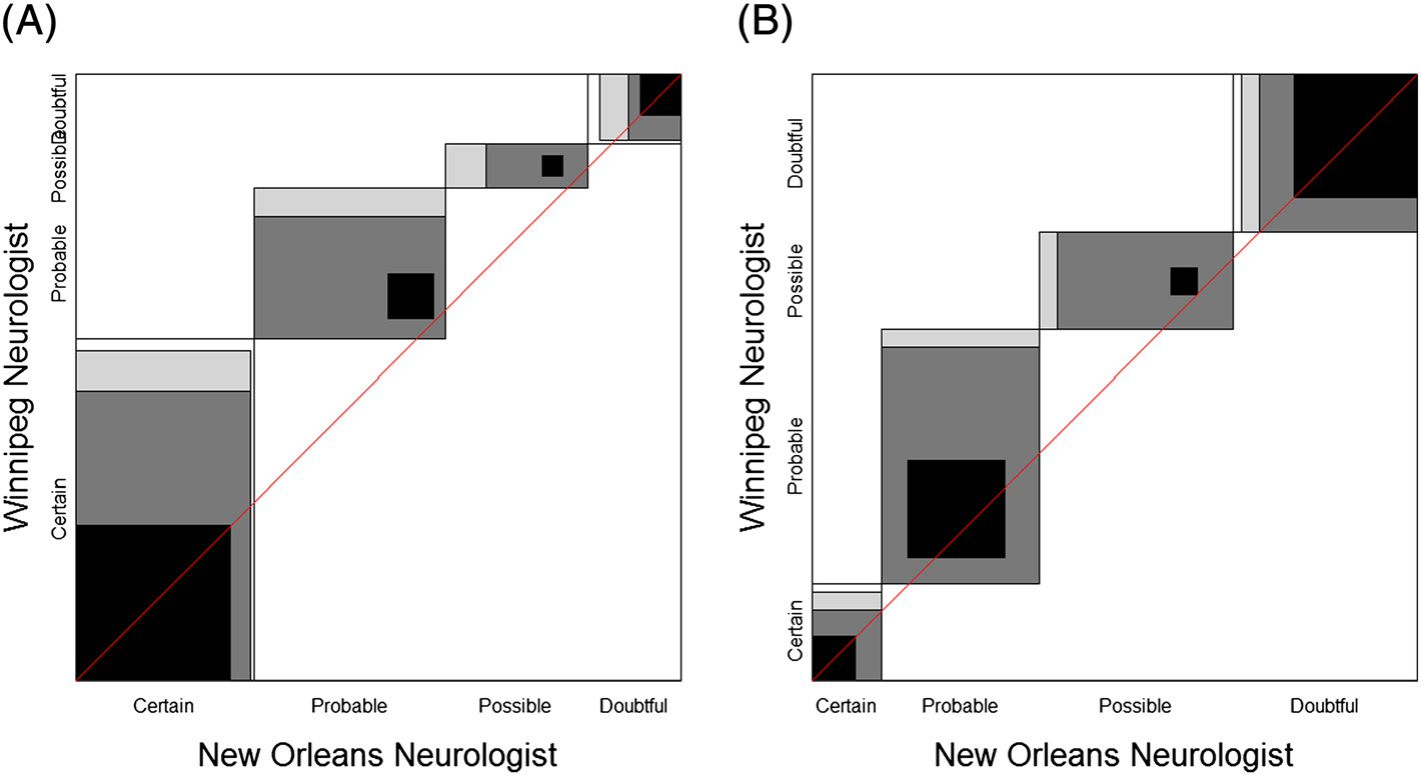
**Agreement charts for comparing multiple sclerosis diagnosis by independent neurologists for (A) Winnipeg patients and (B) New Orleans patients [Westlund &Kurland (1953)].**

In Figure 1 we first note that the Winnipeg neurologist has a bias towards ‘certain’ and ‘probable’ since the ‘path of rectangles’ is above the diagonal line of no bias in both charts. Furthermore, the Winnipeg neurologist tends to be even more biased towards the first two categories for his/her patients (Figure 1A), leading one to guess that the masked evaluation of patients was not truly masked. Thus, examination of the agreement chart can help uncover issues and patterns of disagreement that affect the reliability and validity of cross-classified data, information not obtained from either the kappa or the B summary statistics.

#### Example 2 – Bangdiwala et al. (1989) cardiovascular disease

**Figure 2:**
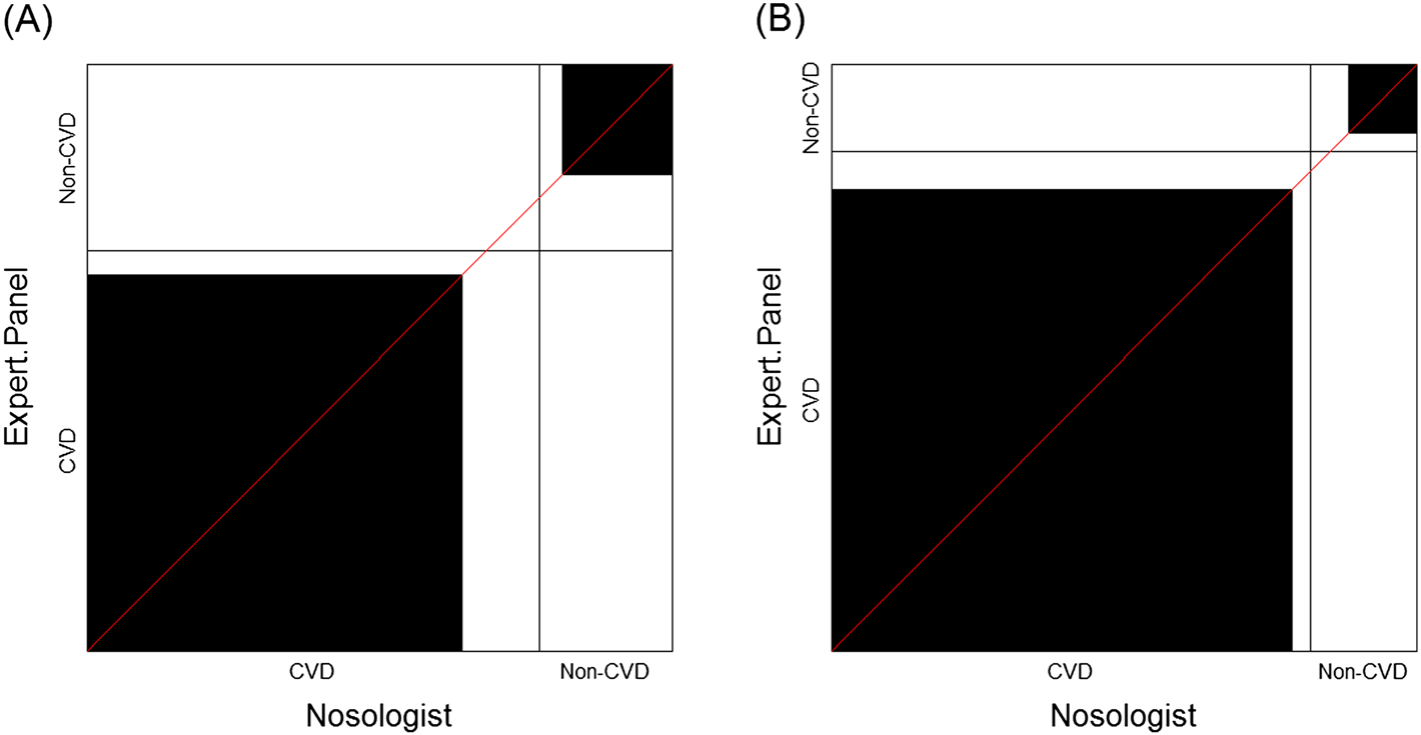
Agreement charts for comparing cardiovascular disease cause of death categories by two independent classification methodologies in the Lipids Research Clinics Program Mortality Follow-Up Study (LRC-FUS) for (A) Elderly (≥ 65 years) deaths and (B) Non-elderly (<65 years) deaths – [Bangdiwala et al. (1989)].

In Figure 2, we first note that there is considerable agreement, driven by the large number of cardiovascular disease deaths in this study sample. While the summary statistics between elderly and non-elderly deaths were comparable, the charts are not. They illustrate a meaningful bias towards CVD attribution in the nosological assessment of the death certificate for elderly subjects, the opposite for non-elderly deaths. The ‘path of rectangles’ for Figure 2(A) lies below the 45° diagonal line, but is above the 45° diagonal line in Figure 2(B).

#### Example 3 – Garrido-Estepa et al. (2010) breast cancer risk categories

**Figure 3:**
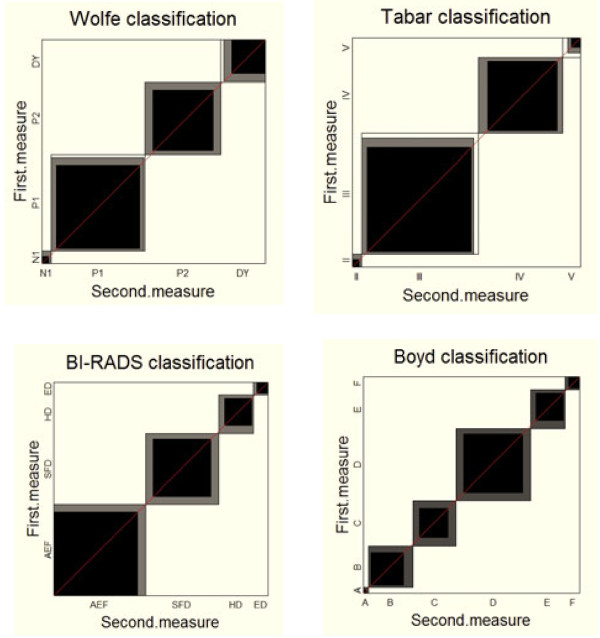
Agreement charts for the comparison of first versus second mearsurements using four different risk classification scales for breast cancer based on mammographic density patterns [Garrido-Estepa et al. (2010)].

The agreement charts in Figure 3 clearly show that agreement is quite high as the rectangles within the unit square are fairly darkened and the ‘one category away’ discrepancy means the shaded portion fills up the rectangles for BI-RADS and Boyd, but not for Wolfe and Tabár. They also show that there is little ‘drift’ bias between first and second measure, since the ‘path of rectangles’ lies along the diagonal. We do note some differences among the scales that are not reflected in the numerical summaries – the preferences for the lowest ‘low-risk category’ for BI-RADS, while Wolfe and Tabár rarely even use the lowest ‘low-risk category’. The four plots in Figure 3 are quite different visually. Note also that Boyd having 6 categorizations while the other scales have 4 is not a hindrance in producing charts that enable the visual comparisons.

## Discussion

When assessing the concordance between two methods of measurement of ordinal categorical data, summary measures are often used. We believe that a picture conveys more information than a single summary measure, and thus this manuscript introduces the ‘agreement chart,’ how it is constructed and interpreted. The objective of this manuscript is to illustrate its use in visually assessing concordance with several example clinical applications as a way to foster its use in clinical applications.

In the examples presented, the information obtained from the charts led to interpretations of the data that were not obtainable form summary measures of agreement. In Example 1 [Westlund & Kurland (1953) [2]], comparing multiple sclerosis classification by two neurologists, examination of the agreement chart uncovered issues and patterns of disagreement that affected the reliability and validity of the assessments between raters. In Example 2 – [Bangdiwala et al. (1989) [7]], the use of the agreement chart uncovered the importance of using a panel of expert cardiologists to classify cause of death as CVD or non-CVD as opposed to relying on nosological assessment from death certificates in elderly versus non-elderly populations. Finally, in Example 3 [Garrido-Estepa et al. (2010) [8]], the agreement charts uncovered differences among the scales that are not reflected in the numerical summaries - identifying ‘drift’ bias between first and second measure, and differences in preferences for the lowest ‘low-risk category’ for some scales.

The main advantage of the agreement chart is that it is a visual representation of agreement, while existing methods to study agreement are either based on summary measures or on modeling approaches. In addition, it is able to provide insight into how disagreements are affecting the comparability of the two raters/observers. There are no other graphs for visually assessing agreement, and it is easily implementable in standard statistical software. The only major disadvantage is that it is limited to ordinal scale variables, since if the categories where on a nominal scale, permuting the order may affect the visual interpretation of agreement.

The utility of the agreement chart is not only for assessing agreement, but also for allowing insight into disagreements. With multiple categories, appropriate shading of rectangles within the agreement chart helps visualize patterns of disagreements. Differences in marginal distributions of ratings by the two observers are visualized by focusing on the ‘path or rectangles’. Various patterns in the path of rectangles may indicate differences in location or variability between the two raters/observers’ preferences for categories.

## Conclusions

Ideally, data should be presented graphically, since “graphics can be more precise and revealing than conventional statistical computations” [10]. Graphs provide a visual impression that no summary statistic can convey, and summary statistics reduce the information to a single characteristic of the data. However, visual impression can be personal and subjective, and not usually reproducible from one reader to another, and graphs occupy more space in a manuscript than a simple summary statistic. Given the choice of measurement scale for the analysis, graphs should be used to complement the summary statistics, not to replace them. However, graphs can be very helpful to researchers as an early step to understand relationships in their data. In this manuscript we used clinically relevant examples to illustrate the additional information provided by the agreement chart when assessing concordance.

## Competing interests

The authors declare that they have no competing interests.

## Authors’ contributions

SIB was involved in conceptualization, literature search, writing and data interpretation of the study. VS contributed in conceptualization, literature search, writing, data analysis and creating charts for the study. All authors read and approved the final manuscript.

## Pre-publication history

The pre-publication history for this paper can be accessed here:

http://www.biomedcentral.com/1471-2288/13/97/prepub
